# Vegetarian Diets During Complementary Feeding: An Overview of Nutritional and Health Features

**DOI:** 10.3390/children12020126

**Published:** 2025-01-24

**Authors:** Marco Brusati, Michela Baiocchi

**Affiliations:** La Volpe e il Canguro, 25062 Concesio, BS, Italy; m.baiocchi@lavolpeeilcanguro.it

**Keywords:** vegetarian diet, vegan diet, complementary feeding, infant feeding

## Abstract

Nowadays, vegetarian diets are present in a solid way in Western countries’ lifestyles. Some families opt for this dietetic pattern for their infants too, during the period of introduction of complementary foods. Many releases have been issued about this subject, with different and often contradictory advice and conclusions. The aim of this work is to provide a comprehensive overview through the analysis of recently published opinions of the implementation of a vegetarian or vegan diet over the course of complementary feeding. The literature agrees about some key points to consider, that is the necessity for the diet to be well-planned, in order to meet energy, macro- and micronutrients requirements, as well as the need to follow the child longitudinally. Also, there is a substantial agreement on the need for fortified foods and/or supplements to be included in the diet. Following these suggestions, most (but not all) of the papers agree that normal growth and development may be achieved. Final considerations, however, are not straightforward to make and more research with better definition of the features of the diet adopted and of supplementation used and long-term follow-up studies are highly warranted.

## 1. Introduction

“Complementary feeding” can be defined as that phase of child feeding characterized by the introduction of new foods into the diet of the infant who, up to that moment, has been exclusively fed with milk, either breast milk or formula milk or both [1]. In this phase, milk is complemented by the use of foods which—according to the preference of the individual family—can be offered through different approaches. For simplicity, two possible non-mutually exclusive alternatives can be identified: (1) At the beginning foods may have a homogenous and smooth texture, offered to the infant by a spoon, usually according to some kind of scheme parents are invited to follow, then progressing to higher consistency and lumpy foods, and then achieving a final diet which resembles the family’s diet at around 1 year of age. This is usually referred to as “spoon-fed traditional weaning” [2,3]. (2) Otherwise, the child can directly and autonomously approach family’s foods, which are still prepared following specific directions in order to be adequate for the development of the infant, nutritionally appropriate, and safe. This approach has been dubbed “baby-led weaning” [3,4]. Even though these two approaches often are presented as far apart, in everyday practice they can present nuances, and families can actually also use a combination of them.

When one should begin complementary feeding is currently still under debate [5]. It can be suggested that “when the infant is ready” may be a valuable policy [6,7]. This apparently simplistic criterion implies that the infant has achieved some skills, which are usually mentioned in relation to baby-led weaning (see below) [8], but which could be considered irrespective of the approach chosen: he/she can show interest in food (or, rather, in what happens during meals); she/he has achieved the control of head and trunk (that is, the infant can sit with little or no support); and he/she can reach out, grab things, and bring them to the mouth. These features usually are apparent around 6 months of age, a time when the infant’s requirements can be no longer satisfied by breast or formula milk alone. On the other hand, it is known that complementary feeding can begin as early as 4 months of age [9]. Therefore, generally speaking, 6 months of age (not before 4 months of age and not beyond 6 months of age) may be considered a proper general starting date, but the timing and approach need to be tailored to the specific features of the individual infant and his family [9,10,11].

Foods introduced can include foods of plant origin along with meat, fish, dairy, and eggs, in the case of an omnivorous diet. When all plant-derived foods are consumed and dairy and eggs too, but meat and fish are excluded, we are talking about a vegetarian diet. This can be further characterized as lacto-ovo vegetarian (LOV) when dairy and eggs are included; lacto-vegetarian (LV) when dairy is included and eggs are excluded; and ovo-vegetarian (OV) when eggs are included and dairy is excluded. When the diet includes plant-based foods and excludes any animal-derived food, the subject is consuming a vegan diet. A clear exception to these claims is the use of human milk (that clearly is, strictly speaking, of animal origin) for infant feeding [12,13,14]. Commonly, the term “vegetarian” is used to indicate collectively both vegetarian diets (LOV, LV, OV) and vegan diets, which, however, can create some inaccuracy in the understanding of the precise diet the person is following or to which the study is referring. Also, “strict vegetarian” is a term used to indicate vegan subjects. In addition to these more common groups, other habits can be identified: pesco-vegetarian or pescatarian (eating fish, not meat), pollo-vegetarian (eating poultry, not other meats or fish), semi-vegetarian or flexitarian (rarely eating meat and fish), macrobiotic (generally following a vegetarian/vegan pattern together with the use of seaweeds, but in some cases eating fish too, and in other cases not eating some vegetables or processed foods), and yet other categories, such as raw-food eaters (consuming raw foods), fruitarians (consuming fruits, nuts, and seeds which can be collected without damaging the plant), and sproutarian (consuming sprouted seedlings) [13,14,15] (see Table 1). Moreover, in real life, there can be middle ways between these different patterns or transitions over time. Finally, “plant-based diets” is used as a broader term to indicate any diet which emphasizes plant products’ consumption with a lower or absent use of animal products [16,17]. Therefore, such a term may refer to a vegan diet, as well as to a vegetarian diet, but even to some omnivorous diets low in animal foods, such as the Mediterranean diet [17]. Consequently, when talking about vegetarian diet(s), the actual food pattern should be further characterized, to better understand what is being talked about. 

An Italian survey [18] based on a sample of 360 families has detected complementary feeding defined as “alternative” in 9.2% of the families: 4.5% as semi-vegetarian (not further specified); 1.9% as lacto-ovo-vegetarian; 0.6% as lacto-vegetarian; and 2.2% as vegan. These figures are in line with EURISPES (Istituto di Studi Politici Economici e Sociali) data reporting that 9.5% of the Italian population claims to be vegetarian (7.2%) or vegan (2.3%) [19] and with data reported by other authors in industrialized countries (4–10% of adults being vegetarian and vegan) [20]. In western countries, the motivations usually identified as the base of the vegetarian choice are the potential health benefits, an ethical choice, a lower environmental impact, religious beliefs [21,22,23], and, specific to the pediatric age, the answer “because the parents eat this way” [23].

In accordance with the Developmental Origin of Health and Disease theory, early life events are capable of influencing the subject not only at the time they happen, but also during different aspects of the development of the subject too, with long-lasting effects, leaving a sort of a mark which may determine the condition of health or disease in different phases of life, until adult age [24,25]. It is clear that diet may be an important driver involved in this role, even though supporting evidence is not fully conclusive yet [26]. 

Therefore, the question that is often asked by parents, as well as by professionals, is whether there are discrepancies between different approaches to complementary feeding or between different dietetic patterns in term of health, present and future. So, the objective of this publication is to analyze recent opinions about the possible nutritional and health benefits or hazards of vegetarian diets implemented in 6 to 12 month-year-old infants and how to address potential critical issues, in order to ensure a healthy present and future. For this purpose, we searched PubMed for publications issued from January 2000 to May 2024, in the English language, pertaining to vegetarian or vegan complementary feeding practices. Key points on which the publications retrieved agree, and, therefore, those which will be analyzed in detail, are the need to ensure the adequate growth and development of the vegetarian/vegan child comparable to those of the omnivorous child; to provide an adequate supply of energy and certain key nutrients, such as protein, ω-3 fatty acids, iron, zinc, calcium, iodine, vitamin D, and vitamin B12; and to pay attention to possible antinutrients. Also, the role of supplementation or fortified food will be regarded.

## 2. Nutritional Considerations

### 2.1. General Directions About Complementary Feeding

General directions about the introduction of complementary foods in infant feeding do not differ depending on the food pattern the family has chosen [12,21,27,28,29]. Bearing in mind the recalled debate about the best timing, in the most recent releases, the age of 6 months is suggested as a general starting point [13,30,31,32,33]. In general, the importance of energy and nutrient density of foods proposed are emphasized; these are important from the early stages of introduction [9,21,29,32,33,34,35,36]. Dietary fats should not be restricted up to 2 years of age [21,29]. 

With regard to the milk the infant should be fed in the second semester of the first year of life, the authors agree that breast milk or formula milk or a combination of them should be continued [12,21,27,29,32]. In healthy-term infants, in the case of a non-breastfeeding vegan family, it is possible to opt for a plant-based formula milk (soy- or rice-based formulas) [8,13,14,20,21,27,28,29,30,32,33,37,38,39,40,41,42,43,44,45], even though some components (namely, vitamin D) could be of animal origin [43]. So-called plant-based milks (that is beverages derived from a plant substrate and resembling animal milk in appearance) can be used in cooking (selecting those fortified at least with calcium and with no added sugars) [32,35,37,43], but not as an alternative for breast or formula milk, as they have a very different nutrient and energy composition compared to animal milks [12,13,21,29,32,37,41,42,43]. 

A recent paper has analyzed the nutritional composition of vegetarian and vegan commercial baby foods launched in the 2017–2021 period worldwide [46]. The authors detected some differences in the comparison between vegetarian and vegan products: vegan 0–12 months formulas, baby cereals, and juices and drinks were significantly higher in saturated fatty acids (SFA) compared to their vegetarian counterparts; vegan 0–12 months formulas, baby cereals, and baby biscuits and rusks were significantly higher in protein; vegetarian 0–12 months formulas and baby cereals were significantly higher in sugar; and, generally, vegan infant foods were significantly higher in iron and calcium content, probably because of fortification [46].

With regards to energy needs, there is no difference depending on the pursued diet, whether it is omnivorous, vegetarian, or vegan [13], and energy requirements can be attained by means of careful food choices (see below for details).

### 2.2. Protein

Proteins and amino acids are a central nutrient in infant and child nutrition because of their function as a structural component of tissues in a body which is physiologically growing. With regard to the origin of proteins in the diet, plant-based proteins have an amino acid composition different from those of animal origin. Usually, legumes are considered to have methionine and cysteine as limiting amino acids (that is in suboptimal proportions to meet the demands of human organism); cereals have lysine and threonine as limiting amino acids; and nuts have lysine, methionine and threonine as limiting amino acids [28,38,47,48,49]. All essential amino acids in similar proportions to those of animal proteins are present in soy and soy products, in lupines, in pseudocereals (buckwheat, amaranth, quinoa), in spinach, and in hempseeds [34]. Therefore, to achieve amino acid adequacy, it is useful to combine different plant-based protein sources, that is to realize the so-called protein complementation, e.g., consuming cereals and legumes, in order to obtain an optimal composition of essential amino acids [28,31,33,41,50,51,52]. In infants and young children, it has been suggested that this association may be realized optimally in the meal or within 6 h of it [34,53], on the basis of Young and Pellett [54]. Indeed, taking into consideration the frequency a child eats (in particular when younger), it can be affirmed that protein complementation takes place spontaneously in a varied diet over the day [22,34,50,53]. Moreover, in some plant-based foods there are components—called anti-nutrients—which can reduce protein digestibility, such as: trypsin inhibitors, present in beans that affect trypsin and chymotrypsin in the gut; phytate, present in several vegetal products that reduce the activity of pepsin and trypsin; and tannins, in legumes and tubers, which make complexes with proteins [55]. Thermal treatment, sprouting, and fermentation can reduce the effect of these compounds, making the digestion of plant protein easier [42,55,56,57]. Given these premises, Messina and Mangles consider an increased protein requirement of 30–35% for the vegan child under 24 months [53]; this figure has beentaken up later by other authors [21,28,50,56]. Bearing in mind that in the age group under consideration the child still consumes breast or formula milk (which are a source of protein, among other things) and given the abundant availability of protein foods in the Western world, many authors agree that the requirements of both proteins and essential amino acids are normally met when the diet is varied and adequate as regards the calories in both forms of vegetarian diet [12,32,34,36,37,40,42,44,47,56].

### 2.3. Ω-3. Fatty Acids

In addition to the known effects of omega-3 fatty acids (ω-3 FA) on inflammatory, immune, and allergic responses, an appropriate intake of ω-3 fatty acids is considered crucial for the correct development of the retina and the brain in the first stages of life [58]. 

The Italian Society of Human Nutrition (SINU) sets the requirement (as Adequate Intake—AI) of long-chain ω-3 fatty acids to 250 mg per day of Eicosapentaenoic Acid (EPA) and Docosahexaenoic Acid (DHA), to which are added other 100 mg of DHA per day in the period 6 to 24 months [59]. The European Food Safety Authority (EFSA) sets the AI for DHA to 100 mg/day for the age range 6 to 24 months, while no AI is set for EPA and DHA in this age group [60]. 

In the first six months of life, breast milk has a content of DHA equal to 0.32 ± 0.22% of total FA [61], which translates into approx. 10 mg/100 mL breast milk, considering a mature breast milk’s FA content of approx. 3 g/100 mL [62], even though exact figures may vary across studies [63]. In Europe, formula milk for the 0–12 months range provides 20–50 mg of DHA per 100 Kcal (that is approx. 13–33 mg/100 mL) [64]. It follows that other sources of ω-3 become important when the proportion of milk consumed begins to decrease. In a vegetarian diet (particularly vegan, as in eggs there may be some EPA and DHA), there are no direct sources of long-chain ω-3 fatty acids, that have therefore to derive from the conversion of α-linolenic acid (ALA) to EPA and DHA. This happens at a rate of 5–10% for EPA and 1–5% for DHA [65,66]. As a result, various authors agree with the need to ensure good sources of ALA (e.g., nuts, flax seeds, chia seeds, hemp seeds, and their oils; little amounts are also present in soybean oil and canola oil) [34,36,37,42,44,50,56]. From a practical point of view, it has been suggested to use ALA-rich oils in 1–2 meals per day [32,34,42]. For the same reason, it is recommended to limit the intake of linoleic acid (LA) (e.g., from sunflower oil and maize oil), so as to maintain an LA/ALA ratio of maximum 4/1 [36]. Also, the intake of saturated fats (tropical oils such as coconut and palm oils) and trans fatty acids (fried and processed foods) is to be limited, since a high intake of these lipids can interfere with an effective conversion of ω-3 FA [18,32,36,42,56]. On the other hand, olive oil (mainly made up of monounsaturated fatty acids) has no effect on the ratio ω-6/ω-3 and can therefore be used, provided that the needs of ALA are first met [32].

Finally, at least in the first two years of life, it is recommended to supplement the child with DHA from microalgae, usually at a dose of 100 mg/day of DHA [12,31,32,34,36,37,42,56,67], and, possibly, with EPA too (which, however, can also result from the supplementation with DHA by retro-conversion [68]).

### 2.4. Fiber

In the analyzed publications it is emphasized that excessive fiber may be detrimental to the growth and development of the child by reducing the calorie density of meals, by interfering with the absorption of macro- and micronutrients, and by leading to early satiety [13,18,28,34]. As regards calcium, some types of dietary fiber may enhance its absorption [28,56,69,70], and on iron absorption, fiber appears to have minor effects [21,29]. For some vitamins, e.g., vitamin B6, the presence of fibers may interfere with their bioavailability, but, as foods rich in fiber are usually rich in several other nutrients too, in a study [71] the fiber intake did not appear to adversely affect the vitamin B6 status of vegetarian women compared to non-vegetarian women. Moreover, fiber fermentation in the large intestine may cause symptoms such as flatulence and diarrhoea [72]. On the other hand, fiber is known to provide health benefits too, through fermentation or a bulking effect and thanks to the presence of accompanying compounds (vitamins, minerals, phytochemicals). For example, certain fiber can act as prebiotics [72]; fermentation by gut microbiota may result in the production of short-chain fatty acids (SCFA), that is butyrate, propionate, and acetate, which have both local effects on the gut and systemic effects on the immune system, the liver, adipose tissue, and other parts of the body [70,72]. Insoluble fiber may impact favourably the intestinal functions, for example, as a bulking agent [70,72]. For infants and children, there is no unanimous consensus about the dietary requirements of fiber [72]. In particular, the EFSA [60] and other institutions [70] do not set recommendations for fiber intake in the 0 to <12-month range.

In many of the analyzed studies there are no specific indications about the type of cereals (refined vs. whole grains) or legumes (hulled or not) or about the characteristics of vegetables and fruits (deprived of most of their fiber or not) to use in the early stages of feeding.

Mexican guidelines from 2016 about complementary feeding, referring to zinc intake, suggest beginning complementary feeding with (among others) whole grain cereals; however, they also note that high amounts of phytates found in whole grains reduce zinc bioavailability [28].

Some releases remain vague, as in Gutierrez et al.’s paper, which suggests “to control the intake of dietary fiber to avoid early satiety and lower intake” without giving more detailed indications [31] or in the 2000s position papers of north American dietetic associations that consider the use of pureed and strained legumes “if necessary” [21,29].

Mangels and colleagues consider the use of whole grains as infant’s chewing abilities improve [13], and whole wheat bread is present in the sample menu for a 9-month-old vegan infant [27], later discussed in the textbook The Dietician’s Guide to Vegetarian Diets [73]. The same authors suggest removing the skin of legumes and, in the first months of complementary feeding, talk about an introduction of vegetables and fruits as strained, mashed, pureed, or as juices [13,27,73]. The position paper by the Spanish Paediatric Association, in the section about complementary feeding, claims that the “use of whole grains should predominate: whole grain bread, brown rice, whole wheat pasta, couscous, millet, corn polenta or quinoa” [37].

Some authors are more prudent about this subject. Baroni et al. suggest to choose refined cereals, hull legumes, strain fruit, and vegetables until 12 to 24 months of age [32,34]. Baldassarre and colleagues recommend for vegan infants up to 12 months of age a diet with little fiber that is rich in refined cereals, hulled legumes, fruit and vegetable juices, and soy products [18]. Andrewski et al. recommend refined grains until the child is 2 years old [38].

Moreover, other authors reserve special treatment for particular situations. The American Academy of Pediatrics’ (AAP) Paediatric Nutrition Manual states that “the sieving or mashing of cereals, pulses, and vegetables that are fed to infants can increase their digestibility, and partial replacement of whole-grain cereals with more highly refined cereals that are lower in fiber can further increase energy intakes and decrease bulk if this is a problem in small children” [74]. Mangels and colleagues speak in a similar way in the case of the child following a macrobiotic diet (which can be particularly bulky and therefore relatively sparse in nutrients and energy) advising grains to be sieved before cooking or to use some refined grains [73].

For later ages, Van Winckel and colleagues suggest for toddlers/preschool children to limit raw unprocessed foods due to lower digestibility and increased chewing effort [41]. For children aged 1–3 years, Weder and colleagues consider a reduction in fiber intake in case the growth of the vegetarian/vegan child is inappropriate [75]. From one year of age, Menal-Puey and colleagues suggest whole grains for children with a vegan diet [76]. In addition, for the vegan child, Messina and Mangels suggest that half of the amount of cereals be from refined cereals and, in the case of early satiety causing low food intake “vegan children may benefit from the consumption of some lower-fiber foods such as refined grains, fruit and vegetable juices, peeled fruits and vegetables, and added fats” [53]. Baroni and colleagues remain cautious about fiber intake up to 24 months of age [32,34].

### 2.5. Phytates

Other compounds to be taken into account for possible antinutritional effects are phytates. These are compounds of inositol with phosphorus (myo-inositol with one to six residues of phosphorus—IP), naturally present in products of plant origin such as whole grains, legumes, seeds, and nuts [55,57,77]. They can have a negative impact on nutrition, with a dose-dependent effect, as they are able to bind bivalent cations such as calcium, iron, zinc, and magnesium. (As for magnesium, the intake from whole grains seems to be able to overcome the effect of inhibition of absorption due to phytate [77].) This makes them insoluble, reducing their bioavailability [20,21,23,28,29,31,44,55,57,65,77,78,79,80]. They can affect proteins and carbohydrates, too [55,80]. On the other hand, phytate can also be beneficial, having antioxidant and anti-cancer activity, activity related to glucose and lipid metabolism, and being able to bind and therefore reduce the bioavailability of some toxic substances, such as lead and cadmium [77,80]. Also, IP3 and IP4 are involved in intracellular signaling [80]. A reduction in the proportion of phytate or the loss of phosphate groups is able to increase the bioavailability of minerals: IP1 and IP2, and perhaps IP3 and IP4 are no longer able to adversely affect the absorption of zinc [77]; IP1 and IP2 do not affect the absorption of iron [77]. Data, however, about the effect of different levels of phosphorylation on calcium are less clear [77]. The fermentation of cereals (e.g., yeast-leavened bread, maize, sorghum) and legumes (e.g., soy beans, chickpeas, beans) can have a positive effect on the bioavailability of minerals through various mechanisms, including the action of endogenous and microbial phytases, which hydrolyze phytate in such a way that the number of phosphate residues and hence the chelating effect are reduced [57,81]. The germination of legumes, oily seeds, and cereals allows endogenous phytases to be activated and produced de novo, thus reducing the phytic acid content [57,77]; soaking legumes and grain cereals before cooking ensures that the phytates leak into the water (which will have to be discarded) and activate the intrinsic phytases, consequently reducing the proportion of phytate in the food [57,77]. The milling of cereals can remove phytate present in the germ and aleurone but causes a loss of minerals, too [57,77,82]. Therefore, overall, these techniques are useful in reducing the potential negative effects related to the presence of phytate in plant-based nutrition [57,77] and can be implemented into a daily diet. In the human organism, phytate can be degraded by the low pH of the stomach, the limited phytase activity of small intestine, the intrinsic phytase of plant origin, and by the phytases from intestinal microbiota [80]. In this regard, a study suggests that a diet rich in phytate (such as the vegetarian diet) shapes the intestinal microbiota, making it more effective in degrading phytate [80].

Currently, there are very different data in the literature about the presence of phytate in foods of plant origin, due to the variability of the actual content in the same food and because of the different analytical methods used [77]. However, the Food and Agriculture Organization (FAO) has recently produced a rich database [83]. It follows that there are still few data on their intake in the child following a vegetarian diet in western countries [82].

### 2.6. Oxalates, Tannins, and Saponins

Oxalates are present in legumes, whole grains, nuts, and some vegetables [55,57,84]. Oxalic acid is capable of binding calcium and magnesium, making them no longer available for absorption [21,29,84]; the effect on iron is not yet fully clear [85], while the effect on zinc seems to be low [57,84]. Soaking, germination, fermentation, and cooking (e.g., boiling) may reduce the content of oxalate, making calcium absorption easier [57,84].

Tannins are a group of polyphenols found in legumes, grapes, and green tea. When consumed in excess, they may have antinutritional properties, by complexing with proteins, therefore inactivating digestive enzymes and reducing protein digestibility [55,56], and by inhibiting iron absorption (see below). Soaking, fermentation, sprouting, and heating may reduce their content [55,57,81].

Saponins are found in grains, legumes, and nightshades. They may negatively affect nutrient absorption by inhibiting enzymes, as well as by binding with nutrients such as zinc, and may lead to the development of a leaky gut [55]. Fermentation can reduce the content of saponins [28,57,81].

On the other hand, tannins and saponins may have potential positive health effects, too, for example, on blood glucose and cholesterol or anti-oxidative, anti-cancer and anti-microbial effects [55,86].

All in all, as stated by the American Dietetic Association (ADA) [69] “a diet of a wide variety of fibre-containing foods also is usually richer in micronutrients”. Therefore, the final effect on the status of the single nutrient can be a less dramatic one on the basis of the mere amount of antinutrients. For example, in an experimental study about bread and young adult men, the first step of an increase in the amount of fiber intake did not influence the balance of iron, magnesium, calcium, zinc, and copper, while a further step had mixed results (broadly, less favorable) [87]. As concerns zinc, it has been reported that the total amount absorbed is larger from whole grains compared to refined ones [53]. As for magnesium, intakes from unrefined cereal-based diets appear to outweigh any inhibitory effect of phytate on magnesium absorption [77].

Thus, from the analyzed papers, the correct intake of fiber and other compounds with possible antinutritional effects still remains a subject of debate. We would like to emphasize the richness in nutrients of foods rich in fiber and the usefulness of making children accustomed from the beginning to the characteristics of whole foods in order to facilitate their consumption at later ages too. In addition, in the event that the child consumes the foods of the family (according to the baby-led weaning approach), the presence also of whole foods, non-hulled legumes, fruits, and vegetables would facilitate meal-sharing. Perhaps the best advice is to customize suggestions according to the characteristics of the individual situation (infant and family), depending also on the specific vegetarian or vegan choice. For example, in the presence of an infant who grows up well and who consumes a wide range of foods, one may be less restrictive and proceed with a consumption of refined cereals next to whole grains; fruits and vegetables may be raw and cooked, or mashed or pureed, removing the most tenacious components; legumes may be hulled or not while checking growth trajectories over time. In the case of a child with more stunted growth or with a more selective diet (still largely based on milk with a small number of other diverse foods), the use of some or all of the measures seen before could be functional to his/her nutrition and growth.

### 2.7. Calcium

Being, together with phosphorus, the main component of the skeleton in the form of hydroxyapatite, a proper calcium intake in the first years of life is essential. As mentioned, the absorption of calcium is limited by the presence of phytate and oxalates [21,29,57,65,84]. Thus, the absorption of calcium from spinach and beets, rich in calcium but also in oxalates, is around 5%; from legumes, almonds, tahin (sesame), and dried figs is 20–25%; from milk and dairy products and soy and soy products is about 30%; and is around 50–60% from oxalate-poor vegetables (e.g., kale, broccoli) [12,21,29,65,86]. The bioavailability of calcium from mineral waters is equal to or greater than that of cow’s milk, fluctuating between 25% and 50% [56,88]. Calcium salts used for food fortification have a variable bioavailability, depending on the single molecule used and the matrix in which it is [56]. Cow’s milk has a high content of calcium, and calcium is also highly available. In cow’s milk, calcium is linked to phosphorylated serine residues of casein. This makes the concentration of calcium high (higher than its maximum solubility), and, as casein is hydrolyzed, it is progressively released, making it available for absorption [89,90,91]. When calcium intakes are reduced, the intestinal absorption of calcium increases, and urinary excretion is reduced [12]. Therefore, as the amount of calcium and its bioavailability in diverse foodstuffs are different, the selection of foods included in the diet may have an impact on the overall calcium status. In addition to cow’s milk and dairy, good sources of calcium for a vegetarian diet, and a vegan diet in particular, are oxalate-poor vegetables (such as broccoli, cauliflower, kale, Brussels sprouts, arugula, watercress); sesame seeds, almonds and their derivatives; soy, tempeh, and calcium-set tofu; dried figs; calcium-fortified plant-based milks and yoghurts; and calcic waters (that is, for Italian legislation, those with a calcium content >150 mg/L—preferably exceeding 300–350 mg/L) [12,31,34]. 

The calcium content of breast milk is around 25–30 mg/100 mL [92,93]. The EFSA set the minimum amount of calcium in infant and follow-on formulas (irrespective to the type of protein the formula contains) to 50 mg/100 kcal [94], which means at least 30 mg of calcium in 100 mL of formula milk. 

The EFSA set 400 mg/day as the AI for infants 6 to 12 months and 600 mg/day as the PRI for children aged 12–36 months [60], and, more recently, to 280 mg/day for 6–12-month infants and to 450 mg/day for 1–3-year-old children [95]. SINU set the requirement of calcium as AI to 260 mg/day for 6- to 12-month-old infants and to 700 mg/day as PRI for the 1 to 3 years age group [96]. Therefore, in the 6–12 month range, most of the calcium requirement is met by milk intake, whether it is maternal or formula [34]. However, in the event that the milk amount falls below circa 500 ml per day, it will be necessary to assess the adequacy of calcium intake from the rest of the diet, and to consider the use of supplements if intakes are reduced [36,42].

### 2.8. Iron

Iron is not only present as a key component of the hemoglobin molecule, but it is also critical for the development and functioning of different cells such as cardiac and skeletal myocytes, hematopoietic, epithelial and immune cells, and for the brain [97]. 

Heme iron in meat and fish has a bioavailability of 15–35% [12,42,56] versus 2–10% of non-heme iron [12,42]. The latter is present in particular in whole grains, legumes including soy and derivatives, in nuts and seeds, but also in vegetables, some spices and aromatic herbs. Its bioavailability is influenced by the presence of inhibitors such as phytate, tannins and fiber [21,23,28,29,44,50,57,65,77,79,85], while the effect of oxalates is still fairly unclear [57,85]. Ferritin in soybeans and other legumes is an easily absorbed source of iron (22–34%) [12]. Overall, in vegetarian diets, an increased iron requirement of 1.8 times that of an omnivorous diet is considered necessary [21,29,31,44,50,56,79,94,98]. This value, however, could be revised downwards by implementing the strategies to increase the bioavailability of iron and is not be applied to the 6- to 12-month age range, as for this age the requirement is already calculated considering a lower iron bioavailability than that of the omnivorous diet of the adult, regardless of the type of diet the child follows [99,100]. To increase the absorption of iron, some strategies may be useful, such as the use in the same meal of sources of ascorbic acid (vitamin C), such as pepper, tomato, parsley, citrus fruits, strawberries, kiwi, papaya, or other organic acids (acetic acid, butyric acid, citric acid, formic acid, lactic acid, malic acid, propionic acid, tartaric acid) or the consumption of carotene or retinol [12,21,23,28,29,31,32,44,45,56,57,65], although some authors question the capability of vitamin C to help obtain an adequate iron status [79]. The soaking of legumes and cereals reduces phytic acid (which, as mentioned, by chelating iron reduces its absorption) through the activation of endogenous phytases, as well as leavening with sourdough, grinding (flour), and cooking [12,21,23,28,29,31,32,65]. However, the long-term effect on iron absorption by inhibitors and facilitators may be less significant than previously thought [12]. In addition, intestinal absorption increases in the long-term in the presence of low iron intake or reduced bioavailability [13,21,29,42,56,65]. Also for this reason, it is possible that the estimates of an iron requirement equal to 1.8 times that of the omnivorous subject are to be revised [12,56]. 

As for breast milk, the iron content is 0.03–0.09 mg/100 mL in mature breast milk [62]. The EFSA set a minimum iron content of 0.6 mg/100 kcal (approx. 0.4 mg/100 mL) in follow-on formula based on intact cow’s or goat’s milk protein or hydrolysed protein, and a minimum of 0.9 mg/100 Kcal (approx. 0.6 mg/100 mL) for formula based on isolated soy protein (ISP) [94]. Wheat germ can be a good source of iron to use in this age group [13,34], as can be the use of fortified foods [13,34]. Possible supplementation is typically made dependent on iron status at blood tests [12,13,42,56,82], even though it is possible to consider it regardless in breastfed children [27,31,41]. 

As for the iron intake in the vegetarian child under one year of age, some authors say that it reaches the reference values even without the use of supplements [15]. 

The iron status of vegetarian/vegan children shows contrasting data depending on the evaluated parameter (e.g., hemoglobin, ferritin, prevalence of iron deficiency anaemia) and the specific study (e.g., setting, age of participants), with values that may be lower than, but more often in line with, those of omnivorous children, both in general and during the period of complementary feeding [15,18,23,56,82]. A study by Taylor et al. is intriguing: children aged 4 to 24 months who are non-meat eaters and meat (plus fish) eaters show a positive nutritional role (blood iron and hemoglobin) for red meat only at 12 months of age, which leads them to affirm that “a vegetarian diet was not generally harmful to iron status” [101].

Some authors point out that an inadequate iron status is a common nutritional problem also in children consuming non-vegetarian diets and, therefore, for vegetarian children it may be prudent to consider the use of fortified foods or iron supplements [79].

### 2.9. Zinc

Zinc is a cofactor of hundreds of enzymes involved, for example, in nucleic acid, protein, and energy metabolism and is a structural component of transcription factors. Moreover, zinc is particularly important for the intestinal mucosa, skin, and immune system. Finally, zinc is essential for normal growth and development [78,102]. 

For zinc, similar considerations apply to those made for iron, resulting in a requirement increase of 50%. According to some authors [50,56], phytate, oxalates and fiber reduce its bioavailability, while the presence of small amounts of animal proteins, amino acids containing sulphur, hydroxyl acids and organic acids, and some procedures (grinding, acid leavening, soaking, sprouting) increase its bioavailability [12,21,23,28,29,31,32,36,44,56,57,65,77,78]. In addition, as for iron, studies also in the first year of life show an adaptation through greater absorption in case of higher demands or lower intakes [12,56]. Also, the study by Miller et al. shows that in infants and young children dietary phytate is not significantly related to zinc absorption [103], although there are still uncertainties about this [77]. Finally, iron supplements may interfere with zinc absorption [78]. 

As regards breast milk, zinc levels in human milk are not related to maternal zinc status [78]. The zinc content is higher in colostrum (approximately 0.8 mg/100 mL), declining rapidly in the first week of lactation and then more slowly, reaching 0.2 mg/100 mL by 2 months, 0.1 mg/100 mL by 6 months, and 0.05 mg/100 mL by 12 months [78] or, more generally, 0.1–0.3 mg/100 mL of mature breast milk [62]. Zinc bioavailability from cow’s milk formula and soy formula is lower than from breast milk [78]. 

The EFSA set the minimum content of zinc to 0.5 mg/100 Kcal (approx. 0.3 mg/100 mL) for follow-on formula based on intact cow’s or goat’s milk protein or hydrolyzed protein, and to 0.75 mg/100 Kcal (approx. 0.5 mg/100 mL) for ISP formula [94]. 

Good sources of this mineral, in addition to milk and dairy products, are legumes (including soy products such as tofu, tempeh, and miso), whole cereals, seeds and nuts, wheat germ [12,31,53], and fortified cereals [40].

If zinc intakes are not considered to meet requirements [21,29,53], or if blood tests show a deficiency, supplementation is recommended [12,42,82]. Other experts suggest supplementing zinc from the beginning of complementary feeding [31]. However, for iron, generally there are no differences in the status of zinc according to the dietary pattern already in the first years of life, and clear pictures of deficits in the vegetarian child are not common [18,21,82,101].

### 2.10. Iodine

Iodine is the mineral present in the thyroid hormones triiodothyronine (T3) and thyroxine (T4), which take part in several of the body’s functions, such as metabolism, growth, development, and reproduction [104]. As a nutrient, iodine is potentially deficient in all diets due to the variability of its presence in foods depending on its content in the soil [76]. 

The iodine content of breast milk fluctuates widely and is affected by the iodine content in the food consumed and possible integration: values can range from 4 to 5 µg/100 mL to 16 to 30 or more µg/100 mL [105,106]; the AAP manual shows values of 15 µg for 100 mL of mature breast milk [62]. The EFSA set the minimum content of iodine to 15 µg/100 Kcal (approx. 10 µg/100 mL) for follow-on formula irrespective of the protein the formula is based on [94]. 

Taking into account an iodine requirement for the 6- to 12-months’ group, expressed as AI, equal to 70 µg/day [107,108], depending on the amount of milk consumed, breast milk and formula milk may contribute to satisfying most of the infant’s iodine requirement [18,32,34,40]. Other authors recommend a supplement of iodine when the vegan infant is no longer exclusively breast-fed [67]. Other sources of iodine in a vegetarian diet are iodized salt (but salt is not recommended at this age), milk and dairy products, eggs, and fortified foods [12,67,108]. With regard to algae as a food, because of the highly variable content of iodine, caution has been suggested if used in infants [35,37], while other authors talk about their use in general with no specific recommendation for infancy [12,36].

A recent cross-sectional study about vegetarian and vegan children aged 0.5 to 18.5 years in the Czech Republic analyzes several parameters of iodine metabolism (iodine intake, iodine in spot urine, markers of thyroid status) and suggests that these children may be more at higher risk of iodine deficiency compared to their omnivorous peers; at the same time, iodine supplementation seems effective in improving iodine status [109]. 

In soybeans, crucifers, and sweet potatoes, there are factors (natural goitrogens) that interfere with iodine uptake by the thyroid gland; however, in healthy people, in the presence of an adequate iodine intake, these foods have not been associated with thyroid dysfunction [12,21,29,86]. In addition, heat treatments are able to inactivate natural goitrogens, thus increasing the bioavailability of iodine [57].

### 2.11. Vitamin B12

Vitamin B12 is a key nutrient for the organism as it is the coenzyme of different enzymes which take part in red blood cell maturation, central nervous system development, and homocysteine metabolism [110].

The authors of the analyzed studies agree that vitamin B12 is not substantially present in foods of plant origin. Some fermented products (from soy, sauerkraut), some algae (nori, spirulina), and some fungi (shiitake) may contain small and variable amounts of vitamin B12, including non-active variants [21,41,51]. Therefore, they cannot be considered reliable sources of this vitamin [12,14,21,29,35,36,42,53,56]. It follows that the vegan subjects’ intakes are generally almost nil (unless they use fortified foods), while those of the vegetarian subjects depend on the amount of dairy products and eggs they eat. Even endogenous production by the gut microbiota, while present, cannot be considered a reliable source of this vitamin [111].

At the moment, when the infant, both vegetarian and (especially) vegan, begins the complementary feeding and therefore reduces the intake of breast milk or formula (from which they obtain a reliable source of vitamin B12 if the maternal intake is adequate), it is necessary to rely on reliable sources of vitamin B12, that is fortified foods or, with greater safety, supplements [8,12,13,23,27,31,32,34,36,37,40,42,45,50,52,56,67,112]. Since the percentage of vitamin B12 absorbed varies according to the dose provided, for supplementation, it is recommended to stay above the reference values. In particular, for this range and up to 3 years of age, it has been recommended to have an intake of 5 µg per day when taken as a single daily dose [32,34,42,50]. Also, thanks to the reduction of intestinal absorption in the presence of high intakes, no adverse effects are reported even in the presence of high consumption [113], even though laboratory vitamin B12 hypervitaminosis has been reported in children with high/frequent supplementation [114].

### 2.12. Vitamin D

Nowadays considered as a prohormone rather than a “simple” vitamin, vitamin D has different effects among which it is possible to mention its actions on the bone, kidney, and intestine to regulate the calcium–phosphorus–magnesium metabolism and on the inflammatory and immune responses [58,115]. 

Regardless of the type of diet, vitamin D requirements, expressed as AI, are estimated to be 400 IU per day in the range 6–12 months [60,116] and 600 IU per day from the first year of age [116,117].

Throughout the first year of life, Italian guidelines on vitamin D recommend supplementing with 400 units per day of vitamin D in the infant born at term [118]. These indications are basically independent of the type of diet, as the status of vitamin D depends more on exposure to the sun rather than on the intake of vitamin D [34,58]. Therefore, even though guidelines may show some small differences in quantity, the duration of supplementation, and the type of milk (breast milk or formula milk), they agree in principle on the need for supplementation [13,27,31,32,34,40,42,44,50,67]. Two forms of vitamin D are available both as supplements and as a component of fortified foods: vitamin D2—ergocalciferol—of non-animal origin (usually produced by ergosterol in fungi and yeasts via UVB irradiation, or obtained from the lichen *Cladonia rangiferina*), and vitamin D3—cholecalciferol—of animal origin (usually from the irradiation of 7-dehydrococholesterol from sheep’s lanolin) or derived from *Cladonia rangiferina* [58,118,119,120,121]. Some authors point out that vitamin D2 has a lower bioavailability [111] or a lower half-life [118], therefore advising an increase in the dose by 1.7 times [111], while others note that both appear effective in maintaining adequate blood levels of 25OH-vitamin D [118], although for higher dosages vitamin D2 seems less effective in improving and maintaining the blood status of vitamin D [12].

### 2.13. Other Possible Nutrients of Interest

Generally speaking, other nutrients that are referred to as potentially critical in a vegetarian/vegan diet at the pediatric age, in some, but not all, publications, are selenium [23,35], vitamin A [9,38,45,46,122,123,124], vitamin B2 [45,122,124,125], and folic acid [8].

## 3. Health Effects

The appropriateness of a diet at the pediatric age can be assessed in different ways, e.g., nutritional adequacy, the impact on growth and development of the child, the impact on general wellbeing, the occurrence (prevention) of pediatric diseases and later diseases in adulthood, health care impact, food costs, and environmental sustainability and ethical considerations [126].

Throughout the development period, and in the first year in particular, energy and nutrients needs per Kg of body weight are higher than the requirements of adults [23,75]. 

From this premise comes that the infant and child may be exposed to the largest risks of deficiencies and consequently inappropriate growth and development [15,76]. This may lead to the assumption that vegetarian and vegan diets are not appropriate in infants and children. Indeed, a prerequisite of any diet is that the diet is well-planned, and therefore that any possible issue is proactively identified in order to be prevented. As a consequence, case reports stemming from improvised or ill-planned food choices [30,127,128] cannot be considered illustrative of vegetarian or vegan diets which, conversely, need to be properly planned to be defined as such [32,111]. As seen above in the analysis of single nutrients, the hazard of shortfalls can be avoided through the knowledge and selection of proper food sources (in quality and quantity), by means of a range of measures to increase the bioavailability of certain substances, and through the thoughtful use of fortified foods and supplements (Table 2). On the other hand, a vegetarian diet may be beneficial thanks to the presence of phytochemical compounds, fiber, a different lipid profile, and/or the absence of potential harmful compounds found, for example, in meat. While in adults there is evidence supporting the benefits of vegetarian diets (e.g., lowering the risk of overweight, hyperlipidemia, metabolic syndrome, cardiovascular disease, cancer, and mortality), only few data are available in children [20].

### 3.1. Growth

Studies on growth parameters can be challenging to interpret because different factors acting at different moments can influence the final result and the conclusions drawn. In this sense, in addition to the sole dietetic pattern, growth can depend on different drivers, such as: the type of milk feeding in the first months of life (breast or formula or a combination of both); the type of animal milk vs. vegetable milks used later; the time point at which the parameter of interest is evaluated (and its possible relationship to final growth); the level of pubertal development; and other lifestyle components (e.g., physical activity)—genetic factors apart. The growth outcome itself can be expressed by different anthropometric parameters (height for age, weight for age, weight for height, BMI, etc.), as an absolute value or as percentiles or z-scores, and compared to different range values. In addition, many studies on this subject are now dated, so they cannot be considered fully representative of today’s reality. Indeed, in their 2009 position paper, ADA suggested that the implementation in infancy and childhood of well-planned vegetarian diets “do not affect final adult height or weight” [29]. More recently, Schurmann and colleagues reported that “The majority of the studies indicated that body weight, body height, and other anthropometric measures of infants, children, and adolescents on vegan or vegetarian diets were in the range of or slightly below the references, or similar to omnivorous control groups” [15]. Of the same year is the position paper by SINU that claims that the growth of LOV children seems similar to that of omnivorous children; vegan non-macrobiotic pre-schoolers are in the normal range, “although they seem to have an initially smaller stature and tend to be leaner than omnivorous children” [56]. The European Society of Pediatric Gastroenterology Hepatology and Nutrition’s (ESPGHAN) position paper about obesity in children notes “The limited evidence available indicates that the growth of LOV children and adolescents is comparable to that of their omnivoric peers. Furthermore, data suggest that VEG children tend to grow in a similar pattern to non-VEG children” [129]. Another release talks about a similar growth rate for “vegetarian/vegan weaning with an adequate intake of essential nutrients” compared to omnivorous peers [8]. The review by Alexy [23] includes data about children from 6 months to 18 years of age, obtained from studies issued from 2018 to 2023. The author concludes that similar anthropometrics are present between vegetarian and omnivorous children, but that there may be a higher prevalence of wasting, stunting, and being underweight in the youngest vegetarians, and that fat mass was lower in vegetarians and vegans in some but not in all studies. This review includes a longitudinal cohort study [130] that can be particular interesting as it is recent (2022) and therefore may represent present-day vegetarian family habits, and it comprises vegetarian (vegetarian + vegan) children aged 6 months to 8 years, therefore including children during the complementary feeding period as well. The authors conclude that no clinically meaningful differences in growth were found (BMI z-score and height-for-age z-score) between vegetarian and non-vegetarian children, although vegetarian diet was associated with higher odds of being underweight (z-BMI < 2SD according to WHO growth charts) [130]. A 2023 meta-analysis showed no differences in height (in cm, after the exclusion of studies with significant age disparity, and z-score) between vegan and omnivorous children and adolescents [125]. The recent cross-sectional studies from the Czech Republic about vegan, vegetarian, and omnivorous children show a higher number of vegan children (median age 2.0 years) with BMI < 3rd percentile [109,114]. The 2024 review by Desmond et al. concludes that, while in vegetarian children anthropometrics markers seems to be comparable to or slightly lower than the reference group, vegan children tend to have lower values of weight, height, and fat mass and a higher risk of being stunted or wasted compared to omnivorous children [131]. This review, in addition to studies already included in previous reviews, comprises a 2021 Italian study that shows statistically significant lower growth parameters (weight at birth, 6 and 12 months of age; length at 12 months; BMI at 6 months) in vegan vs. omnivorous children, while no statistically significant difference is detected between LOV/LV vs. omnivorous and LOV/LV vs. vegan [30]. The authors conclude that the “length-weight growth during the first year of life has been harmonic and physiological regardless of the type of planned diet” [30]. 

Therefore, from the studies reported, it seems that growth may be similar for omnivorous, vegetarian, and vegan children, but a tendency towards lower values can be associated with vegetarian and vegan diets. These insights should not be seen as a reason to discourage vegetarian diets tout court; instead, they should serve as a warning for healthcare staff to correctly advise parents and monitor children over time, and for parents to properly implement what has been suggested that they do.

### 3.2. Bone Health

Particular attention should be paid in children to bone health, which is influenced by calcium, vitamin D and protein intakes, dietary acid load, and the level of physical activity, as the peak of bone mass is achieved during adolescence and a high peak bone mass is protective against later osteoporosis and fractures [23]. Although available evidence at the pediatric age is scarce, it highlights the possibility of lower bone mineral content and density in vegetarian (particularly vegan) children vs. omnivorous ones [23,125,131]. These results suggest that more attention to this aspect has to be paid at the individual level and more information about long-term outcomes in particular is desirable. 

### 3.3. Cardiometabolic Status

In the extensive review about the vegetarian diet at the pediatric age, Schurmann et al., because of the limits in the analyzed studies, conclude that “the existing data do not allow us to draw firm conclusions on health benefits or risks of present-day vegetarian type diets on the nutritional or health status of children and adolescents in industrialized countries” [15]. Yet, they point out as a “tentative conclusion” that “most studies presented here did not show detrimental effect of vegetarian diets in children but even pointed to beneficial health outcomes compared to omnivore diets, such as favourable lipid profile, antioxidant status, or dietary fiber intake as well as tendencies towards a lower risk of overweight” [15]. In this direction goes the German VeChi Diet Study too, which detects a higher prevalence of the risk of being overweight, overweight, or obese in the group of omnivorous children vs. vegetarian and vegan children [75]. Indeed, with regard to this aspect, the vegetarian diet showed to be able to prevent overweight/obesity as well as being considered a valuable alternative to promote weight loss [132]. Still, in two recent reviews, blood lipids appeared more favourable in vegan children compared to vegetarian or omnivorous ones, but not in all studies [23,131] and the meta-analysis by Koller et al. showed lower low-density lipoprotein (LDL) in vegan children compared to omnivorous ones [125]. Overall, therefore, a plant-based diet commenced in childhood may be considered as a useful primary prevention strategy of cardiovascular disease [133,134], even though more specific research of the pediatric age is undoubtedly warranted.

### 3.4. Hematochemical Data

Some authors state that there are no differences in hematochemical data between vegetarian and omnivorous children [8,130], while others report possible differences in lipids (total cholesterol, high-density lipoprotein (HDL) and LDL, DHA), vitamins (B12, D, B2, folate), serum levels of certain amino acids (valine, lysine, leucine, isoleucine), iron status, and iodine status [125,131]. The paucity of data does not allow firm conclusions to be drawn, but this information can be a reference for knowing which aspects of diet, blood, and urine chemistry to monitor the most. 

### 3.5. Eating Habits

It can be assumed that the consumption of a vegetarian diet from a young age may contribute to the establishment of lifelong healthy eating habits [21,29,37,44].

### 3.6. Final Remarks About Health 

It is hard to summarize in brief the positions expressed in the analyzed publications as often they need their own correct contextualization. For example, Gutierrez and colleagues, acknowledging the specific context of Chile (namely, that following an adequate vegetarian diet may be challenging, because of limited access to fortified foods, supplements, and nutritional counselling), recommend a balanced omnivorous diet in the pediatric population, but, at the same time, they call on health professionals to support parents and children who, properly informed, choose these kinds of diets [31]. Also, the consensus from the Latin American Society for Pediatric Gastroenterology, Hepatology and Nutrition states that “vegan, raw vegan, and macrobiotic complementary feeding practices are not recommended” [39], basically because of a possible macro- and micronutrients deficiency. They also state that “Vegan diets, with the appropriate supplements, translate into adequate growth and development” [39]. 

With this in mind, we can affirm that most of the publications state that well-planned vegetarian and vegan diets are appropriate during infancy, too, namely, during complementary feeding [8,12,13,21,29,32,34,35,36,38,44,46,50,56,67]. Other authors state the same specifically for the vegan diet [27].

The ESPGHAN [9] agrees about the feasibility of vegetarian and vegan diets during complementary feeding but stresses the extreme caution with which a vegan diet has to be managed.

Other authors consider the vegetarian choice possible but are more uncertain about vegan complementary feeding [37,40,41,52]. 

Some consider the vegetarian diet as “a feasible alternative if implemented with supervision by a specialist, especially during vulnerable periods of life”, while “vegan diets are not recommended at any age” [33]. Others consider vegetarian complementary feeding possible, while vegan complementary feeding is discouraged [18]. And some express views against a vegan diet during complementary feeding [39], or “for children or adolescents of any age” [51], or for “infants, children, and adolescents” [42]. 

It has also been suggested that, for the pediatric age, there should be a sort of a ranking of inappropriateness, that is, in increasing order: flexitarians, lacto-ovo-vegetarians, lacto-vegetarians, pescatarians, vegans, and macrobiotics [20]. Therefore, the author discourages vegan (and macrobiotic) diets “in young children”, even though he states too that “data for children are limited which precludes a formal conclusion” [20].

A recent Italian publication states that “vegetarian and vegan diets cannot be recommended during the CF period” [123].

Other authors state that existing data do not allow firm conclusions to be drawn about vegetarian diets in infants and children [15,135,136] or do not clearly speak for or against [28,30,45,122,124,131,137].

Finally, it is worth considering that possible deviations from the theoretical requirements, as well as potential deleterious health effects, can be detected when children follow omnivorous diets too. Therefore, it has been suggested that the focus of attention should be shifted onto the adequacy of the diets, vegan or omnivorous [126].

## 4. Caring for the Vegetarian Child

Basically, all the papers stress the need for the child and his family to be followed by qualified personnel, able to advise and support the family and properly plan the diet and the use of supplements [8,9,13,20,23,27,28,30,31,33,34,36,39,40,44,45,50,51,52,122,124,126,137]. Therefore, families who choose this type of diet are invited not to do so on their own, but to refer to a specialist who will follow them over time. At the same time, families can play an active role by informing and training properly, dealing with the specialist on whom they rely, and following his/her advice over time. However, it is fair to stress that parents should be knowledgeable about infants’ and children’s nutrition regardless of the diet they mean to implement. 

Interestingly, a recent Italian study about vegan children reported that “almost all the parents asserted that they had looked at data regarding the nutritional adequacy of vegan diet before opting to raise their offspring as vegans”, especially from healthcare professionals and scientific websites [22]. This shows that, at least as far as the parents recruited in the study are concerned, this food choice was an informed choice, with a knowledge base on the subject. For the internet, a bivalent role is conceivable: on the one hand, it can induce individuals to follow diets “advertised” and poorly implemented [74]; on the other hand, it can become an important source of information about possible positive and negative features of vegetarian diets [23,44,74].

The monitoring of the child over time should concern growth parameters (height, weight, head circumference—for gender and age), psychomotor development, the adequacy of the diet, including the use of supplements and nutritional behaviour [8,13,14,18,20,22,23,28,30,31,40,41,45] and, at least in individual cases, if in doubt of deficiency, an assessment of the status of some parameters through blood and urinary tests [31,45] that could even be conducted routinely [14,18,40,42,51,56]. 

Therefore, in the light of the recommendations analyzed, in everyday practice we suggest considering the following key points in the diet of a vegetarian or vegan infant (Table 2) and we propose the following investigations in order to check growth, development, and nutritional status of the infant (Table 3). 

We suggest that a well-planned vegetarian diet has all of the following characteristics:

- It is a diet based on plant-derived foods (see Table 1 for different vegetarian eating habits);
- It is a diet meeting the energy and nutritional needs of the single subject, also by the use of fortified foods and/or supplements;
- It is a diet planned together with the family, that pays attention to children’s and infants’ requests, suggesting possible modifications to their demands (or what they are currently doing) and that advises about the wise choice and preparation of food and meals. The final aim may be to assure that the family realizes which are the features of a healthy diet, so that caregivers will be able to make responsible food choices on their own. The planning in itself may consist of the realization of a detailed and tailored menu containing the types of foods to consume at each meal or snack, even with quantities, or to more general (but still practical) indications about how to meet needs (for example calcium requirements, that is the use of calcium-set tofu, vegetable milks enriched with calcium, calcium waters, etc.);
- It is a diet longitudinally followed by pediatric and nutritional staff.

## 5. Limitations, Knowledge Gaps and Future Directions

This review has some limitations. First, we analyzed opinions published in the last 24 years (2000–2024). Even though this has narrowed the number of available studies, it has also generated more up-to-date information (also in view of variations in the general recommendations regarding early-life nutrition, the focus of extensive research in recent years) that is in line with current food availability (e.g., the presence of commercial products and consumer habits) and vegetarian population awareness of balanced vegetarian diets, so that conclusions can be more easily applied to the current context. Likewise, although the exclusion of data deriving from low-income settings is a limitation, it is our opinion that including populations with very different socio-economic background would risk making conclusions representative of no situation, neither high-income settings nor low-income settings. Similarly, we considered only healthy infants without taking into account pathological situations which may appear in the first year of life, such as metabolic disorders, immune diseases or surgical conditions. Certainly, these situations deserve consideration in further studies. Relying almost only on peer-reviewed publications, we excluded a bulk of publications (that can be found elsewhere [126]). With the exception of the two handbooks referenced (one of which was edited by the American Academy of Pediatrics), we used this approach to gather evidence that had passed the rigorous exam of peer-reviewing and official publication, thereby excluding personal or subjective ideas or interests. 

The available literature has some limits too. Many of the papers analyzed include studies that gathered data on vegetarians and vegans without distinction, in some cases including other dietary regimes (e.g., macrobiotics), which does not make it entirely possible to separate data regarding different dietary patterns. Often, in the studies, the presence or absence of supplementation or the use or not of fortified foods is not explained, and this may have an important impact on the final results and therefore on the conclusion that may be drawn. Even the presence of a person of reference (pediatrician, nutritionist, dietitian) that cares for the nutrition of the child is not assessed, and it has been stressed how much this factor can be decisive in the adequacy of the diet. Again, some studies gather insights from subjects of different ages (from infancy through childhood to adolescence), without age stratification. In others, the number of subjects analyzed is small; biases are possible (e.g., associated with the voluntary participation to a study or due to other differences in the lifestyles of participants); outcomes analyzed are often different, which makes it difficult to summarize data into a single opinion; and many studies are cross-sectional, and therefore it is not possible to have long-term information or to draw conclusions on cause–effect relationships. In addition, some studies were conducted in the 1980s and 1990s, and, therefore, in the presence of a commercial availability of plant-based foods different from the current ones. Also, in the control groups, the features of the diets in studies of 30 to 40 years ago could be different from the current ones. All this makes it difficult to interpret the results and draw general conclusions about, for example, the intake of certain specific nutrients. Moreover, much information is derived from the estimation of intakes, while data on nutritional status are less frequent, e.g., from blood tests, urine tests, or other investigations. Therefore, in future studies, a more accurate assessment of the actual intakes, supplementation use, and nutritional status is essential to better understand the adequacy of the vegetarian diet in infants and children. The use of ultra-processed plant-based foods remains a topic to explore. Long-term studies will be essential to correlate the diet effectively followed (e.g., vegetarian or vegan, or other patterns—including the detailed use of supplements) with health outcomes, with particular reference to the length/height and weight growth, fat mass/lean mass, BMI, pubertal development, psychomotor development, bone health, and the possible prevention of non-communicable diseases. From this, it will be possible to stem food-based dietary guidelines specific for the pediatric age and the food pattern chosen, and based on the regional retailer food availability. Many insights about these matters will be available in the near future through ongoing studies (e.g., the one form Umea University [138]). Also, the allergic potential of plant-based complementary feeding, the microbiota status, and costs need further clarification. 

Finally, the data are derived mainly from studies of children of industrialized countries, where it is possible to assume that no limitations to the access to food are present, and therefore that there are no limitations in the construction of an adequate diet. Thus, the conclusions presented cannot be transferred directly to situations where access to food is less secure, situations in which new insights are needed. As for the choice of excluding the macrobiotic diet from the analysis, if it has left out a subset of subjects with a vegetarian eating pattern, this decision comes from the observation that the food choices of macrobiotic subjects can be very different from one to another, in some cases including fish and derivatives or excluding supplementation, and, therefore, they may not be fully comparable to the choices of a vegetarian or vegan subject. Last, in the context of a plant-based diet, it will be intriguing to analyze data according to the different approach to complementary feeding implemented, that is puree spoon-feeding, baby-led weaning, and everything that lies in-between in real-world family practice, as well as the use of unprocessed foods (e.g., home-prepared grains and legumes) vs. processed foods (e.g., industrial baby purees, packaged products, and meat and dairy substitutes). 

## 6. Conclusions

The analysis of the recent available literature has detected positions that are not always aligned, both in terms of the possibility of implementation and of health effects. These differences can broadly be explained in the light of the concept of the “properly planned diet”. We affirm that a vegetarian or vegan diet is not simply a diet that excludes animal products or animal-derived products, but rather it is a diet that (1) includes a wide variety of plant-based foods; (2) uses fortified foods and/or supplements, as needed; (3) meets energy and nutritional needs; and (4) is monitored over time by proper personnel. All this makes feasible, even in infancy and childhood, a diet that, if it was simply a do-it-yourself diet, may result in deficiencies and failure to thrive, as extensively highlighted by the literature and media, too. These potential issues should not restrain the correct implementation of these diets. Rather, as for any diet, they are a warning to rely on qualified personnel, able to assess individual requests and needs and able to tailor a food plan appropriate to the individual’s needs, and then follow the child and the family over time.

In conclusion, even though the available evidence is incomplete, and therefore no firm conclusion can be drawn, and the background knowledge needs to be improved through the addition of new studies, we suggest that the picture that emerges from the current evidence is that vegetarian and vegan diets, when properly planned, implemented and monitored, are safe even in the delicate stage of life that is complementary feeding, and may even be useful as a strategy to ensure better health for the subject, society, and the planet.

## Figures and Tables

**Table 1:** Simplified classification of the most frequent dietetic patterns according to foods’ use.

Nomenclature	Animal Foods					Plant Foods
	Meat	Fish	Eggs	Milk and Dairy	Honey	
Omnivorus	✔	✔	✔	✔	✔	✔
Flexitarian (or semi-vegetarian)	✔✘	✔✘	✔✘	✔✘	✔✘	✔
Pescatarian (or pesco-vegetarian)	✘	✔	✔	✔	✔	✔
Vegetarian						
Subcategories:						
- lacto-vegetarianism (LV)	✘	✘	✘	✔	✔	✔
- ovo-vegetarianism (OV)	✘	✘	✔	✘	✔	✔
- lacto-ovo-vegetarianism (LOV)	✘	✘	✔	✔	✔	✔
Vegan	✘	✘	✘	✘	✘	✔
Pesco-vegetarian or pescatarian	✘	✔	✔	✔	✔	✔
Pollo-vegetarian	poultry	✘	✔	✔	✔	✔
Macrobiotic	✘	✘✔	✘	✘	✘	✔ some may avoid some vegetables
Raw-food eaters	✘	✘✔	✘	✘✔	✘✔	✔
Fruitarians	✘	✘	✘	✘	✘	fruits, nuts, seeds which can be collected without damaging the plant
Sproutarian	✘	✘	✘	✘	✘	sprouted seedlings such as grains, vegetables, fruits

✔, included in the diet; ✘, not included in the diet; green text, included in the diet; red text, not inclued in the diet.

**Table 2:** Possible remarks on the diet of vegetarian/vegan infants.

Key Points	Main Plant Food Sources	Possible Suggestions
Type of milk	- Soy- or rice-based formulas	- Exclusive breast feeding or formula feeding up to 6 months of age, then continued during introduction of complementary foods - In the case of use of formula, use cow’s milk-based formulas or soy-based formulas or rice-based formulas - Plant-based milks/beverages cannot be used as alternatives to breast or formula milk, but can be used in cooking
Energy	- Grains - Legumes - Nuts and seeds - Avocado - Fruit and dried fruit - Oils - Plant-based milks (depending on the single product)	- Adequate supply should not be an issue in Western countries and is the base to meet nutritional requirements
Protein	- Grains - Legumes - Nuts and seeds	- Adequate supply should not be a problem in Western countries - Protein complementation occurs spontaneously within the day
Ω-3 FA	- Nuts and its oil - Flax seeds and its oil - Chia seeds and its oil - Hemp seeds and its oil - Canola and its oil - Soybean oil	- Increase ALA sources - Reduce LA sources - Supplement with 100 mg DHA until 24 months of age
Antinutrients	- Grains - Legumes - Nuts and seeds - Vegetables - Fruits - Tea, cocoa	- Soaking, sprouting, fermentation, cooking, as means to decrease the possible effect of antinutrients - The amount of fiber is a matter of discussion: reduce the amount of fiber in any case vs. only in selected cases, such as poor growth
Iron	- Legumes - Whole grains - Nuts and seeds - Green leafy vegetables - Wheat germ	- Consider adding a source of vitamin C to increase non-heme iron absorption - Supplement with iron in breast-fed infants or according to blood tests
Zinc	- Legumes - Whole grains - Nuts and seeds - Wheat germ	- Include good sources of zinc - Supplement from the beginning of CF, or in case of low intake, or abnormal blood test
Calcium	- Broccoli, cauliflower, kale, Brussels sprouts, arugula, watercress - Sesame seeds, almonds, and their derivatives - Soy, tempeh and calcium-set tofu - Dried figs - Calcium-fortified plant-based milks and yoghurts - Calcium-rich waters	- Once breast or formula milk intake decreases, consider good sources of calcium; use supplement if in doubt of shortcomings
Iodine	- Seaweeds - Iodized salt	- Once breast or formula milk intake decreases, consider other sources of iodine already present in the diet or supplementation - Avoid the use of table salt (iodized or not) - Seaweeds use is discouraged
Vitamin D	- Basically absent	- Supplementation for all infants irrespective of the diet followed
Vitamin B12	- Basically absent	- Supplementation for all infants irrespective of the diet followed

Ω-3 FA, omega-3 Fatty Acids; ALA, Alpha-Linolenic Acid; LA, Linoleic Acid; DHA, Docosahexaenoic Acid; CF: complementary feeding.

**Table 3:** Suggested checks for children following vegetarian/vegan complementary feeding.

Pediatric and Dietetic Investigations		Blood or Urine Test	
Type of Examination *	Timing	Type of Examination *	Timing
Growth (for age and sex), according to validated percentile/z-scores: - weight - height - head circumference	At each well-child visit (i.e., every 2 months between 6 and 12 months)	Complete blood cell count	At the beginning of CF or at the first contact with the family and then at 1 year of age
Developmental milestones	At each well-child visit (i.e., every 2 months between 6 and 12 months)	Blood iron, Ferritin	“
Diet followed:		Blood zinc	“
- assess the knowledge base of the family about infant nutrition and vegetarian diets;	At the time of the first contact with the family		
- define what kind of foods the infant and his/her family are consuming and what avoiding (e.g., by 24 h food recall or food frequency questionnaire)	At each well-child visit (i.e., every 2 months between 6 and 12 months)		
Use of supplements and/or fortified foods	At each well-child visit (i.e., every 2 months between 6 and 12 months)	Vitamin B12, Folic acid, Homocystein, Holo-transcobalamin II, Methylmalonic acid (blood or urine)	“
		Total protein, Albumin	“
		Creatinine	“
		Glycemia	“
		Triglycerides, Total cholesterol, HDL, LDL	“
		25-OH vitamin D	“
		TSH	“
		PT (as a proxy for vitamin K status)	“
		Chemical-physical urinalysis	“
		Iodine in spot- or 24 h-urine	“

CF, complementary feeding; HDL, High-Density Lipoprotein; LDL, Low-Density Lipoprotein; TSH, Thyroid Stimulation Hormone; PT, Prothrombin Time; * Additional investigations can be applied according to the single situation and the results of these tests.

## Data Availability

Data are available by consulting the respective references.

## Abbreviations

The following abbreviations are used in this manuscript:

Ω-3 FA	Omega-3 Fatty Acids
AAP	American Academy of Pediatrics
ADA	American Dietetic Association
AI	Adequate Intake
ALA	Alpha-Linolenic Acid
CF	Complementary feeding
BMI	Body Mass Index
DHA	Docosahexaenoic Acid
EFSA	European Food Safety Authority
EPA	Eicosapentaenoic Acid
ESPGHAN	European Society of Pediatric Gastroenterology Hepatology and Nutrition
EURISPES	Istituto di Studi Politici Economici e Sociali
HDL	High-Density Lipoprotein
IP	Inositol Phosphate
LA	Linolenic Acid
LDL	Low-Density Lipoprotein
LOV	Lacto-Ovo Vegetarian
LV	Lacto Vegetarian
OV	Ovo Vegetarian
PRI	Population Reference Intake
PT	Prothrombin Time
SCFA	Short-Chain Fatty Acids
SINU	Società Italiana di Nutrizione Pediatrica
TSH	Thyroid Stimulation Hormone
WHO	World Health Organization
